# Myocardial calcification: case reports and a systematic review

**DOI:** 10.1093/ehjimp/qyae079

**Published:** 2024-07-30

**Authors:** Takashi Kido, Kazuki Tanimoto, Takuji Watanabe, Masaki Taira, Jun Narita, Hidekazu Ishida, Ryo Ishii, Takayoshi Ueno, Shigeru Miyagawa

**Affiliations:** Department of Cardiovascular Surgery, Osaka University Graduate School of Medicine, 2-2, Yamada-Oka, Suita, Osaka 565-0871, Japan; Department of Cardiovascular Surgery, Osaka University Graduate School of Medicine, 2-2, Yamada-Oka, Suita, Osaka 565-0871, Japan; Department of Cardiovascular Surgery, Osaka University Graduate School of Medicine, 2-2, Yamada-Oka, Suita, Osaka 565-0871, Japan; Department of Cardiovascular Surgery, Osaka University Graduate School of Medicine, 2-2, Yamada-Oka, Suita, Osaka 565-0871, Japan; Department of Pediatrics, Osaka University Graduate School of Medicine, 2-15, Yamada-Oka, Suita, Osaka 565-0871, Japan; Department of Pediatrics, Osaka University Graduate School of Medicine, 2-15, Yamada-Oka, Suita, Osaka 565-0871, Japan; Department of Pediatrics, Osaka University Graduate School of Medicine, 2-15, Yamada-Oka, Suita, Osaka 565-0871, Japan; Department of Cardiovascular Surgery, Osaka University Graduate School of Medicine, 2-2, Yamada-Oka, Suita, Osaka 565-0871, Japan; Department of Cardiovascular Surgery, Osaka University Graduate School of Medicine, 2-2, Yamada-Oka, Suita, Osaka 565-0871, Japan

**Keywords:** myocardial calcification, sepsis, myocarditis

## Abstract

**Aims:**

Myocardial calcification is an unusual condition in which excess calcium is deposited in the myocardium. Herein, we report two cases of myocardial calcification from our clinical experience. Furthermore, we conduct a systematic review to examine the clinical course and associated pathologies of myocardial calcification.

**Methods and results:**

This systematic review was registered in PROSPERO (CRD42023463285). PubMed and Scopus were searched according to the following inclusion criteria: (i) case reports or case series describing patients with myocardial calcification; (ii) diagnosis of myocardial calcification by computed tomography (CT); (iii) adequate description of patients, including their chief complaint, medical history, evaluations, and treatments; and (iv) publication in English. Among the 75 patients, 24 had sepsis, 14 had myocarditis, and 37 had other pathologies. The mortality rate was 33% for patients with sepsis, 14% for patients with myocarditis, and 11% for patients with other pathologies. Follow-up CT findings beyond 2 years were reported in six patients, showing that the CT findings of myocardial calcification persisted but subsided over time. Autopsy was performed in seven patients, and extensive interstitial fibrosis and collection of inflammatory cells were observed in patients with myocarditis, sepsis, and ischaemic heart disease.

**Conclusion:**

While various medical conditions can cause myocardial calcification, accompanying conditions commonly reported with myocardial calcification were sepsis and myocarditis. The CT findings of myocardial calcification tend to regress over time if the underlying disease can be treated.

## Graphical Abstract

**Graphical abstract:**
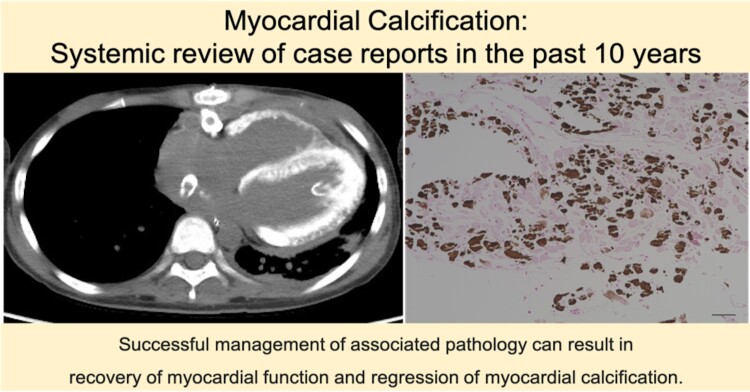

## Introduction

Myocardial calcification is an unusual condition in which excess calcium is deposited in the myocardium. This condition is extremely rare and complicated by myocarditis, ischaemic heart disease, sepsis, and abnormality of calcium metabolism.^1^ In most cases, myocardial calcification is incidentally detected by computed tomography (CT), and this is considered to be a sign of cardiac dysfunction and poor prognosis. The rarity of myocardial calcification limits the feasibility of cohort studies. Existing systematic reviews are small in scale, include old literature, and evaluate only certain causative conditions such as sepsis.^2^ Combining recent studies to include all causative conditions in a systematic review will enhance our understanding of the clinical courses of myocardial calcification and provide data to counsel patients and their families. Herein, we present two cases of myocardial calcification and describe the clinical characteristics of this condition in a systematic review covering the past 10 years.

## Methods

This systematic review was registered in PROSPERO (CRD42023463285) and is reported in accordance with the Preferred Reporting Items for Systematic Reviews and Meta-Analyses guidelines.^3^

## Search strategy and inclusion criteria

On 18 October 2023, we conducted a comprehensive search of PubMed and Scopus using the following search terms: (‘myocardial calcification’ or ‘ventricular calcification’) or (‘myocardium’ and ‘calcification’). The search was restricted to articles published after 2013. The studies were required to meet the following criteria to be included in our review: (i) case reports or case series describing patients with myocardial calcification; (ii) diagnosis of myocardial calcification by CT; (iii) adequate description of patients including their chief complaint, medical history, evaluations, and treatments; and (iv) publication in English.

## Study selection

After the search was completed, two authors (T.K. and K.T.) independently reviewed each abstract for consideration of a full-text review. The same two authors independently reviewed the full text of the articles for inclusion in the systematic review. If the full text was not available online, we sent an email to each author requesting the full text. If we had not received a response after 2 weeks, we sent another email. If we had not received a response after another 2 weeks, we excluded case reports. Once the final articles were selected, data were extracted by one author (T.K.) and validated by another (K.T.). The following criterion was used for exclusion of studies: case reports not showing heart images by CT. Articles that were excluded after the full-text review were recorded with reasons for exclusion. The complete searches are given in Supplementary data online, *Data S1*.

## Interpretation of data

Data were collected on patients’ demographics, chief complaint, medical history, timing of CT showing myocardial calcification, cardiac function, estimated aetiology of myocardial calcification, treatment, and morbidity and mortality. Treatment information was collected on the requirement for intensive care treatment, extracorporeal membrane oxygenation (ECMO), and renal replacement therapy (RRT). Histopathological data were collected if autopsy was performed in deceased patients.

## Quality assessment

Because of the low level of evidence and high risk of bias, the case reports and case series did not undergo a quality assessment.

## Case presentations

### Case 1

**Figure 1:**
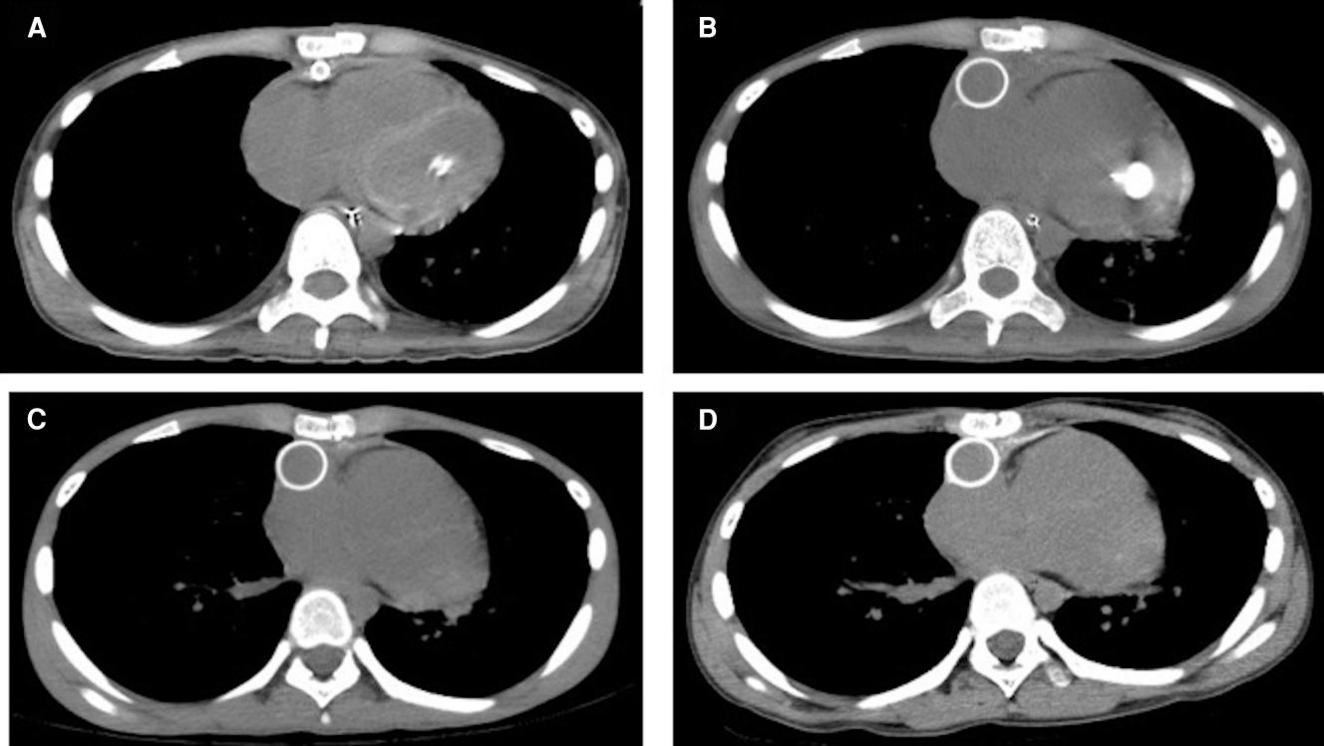
Case 1. A time course of myocardial calcification on CT at (*A*) 3 months, (*B*) 4 months, (*C*) 7 months, and (*D*) 14 months after admission. An annular dense shadow appeared on the LV wall 3 months after admission, but it regressed during follow-up.

**Figure 2:**
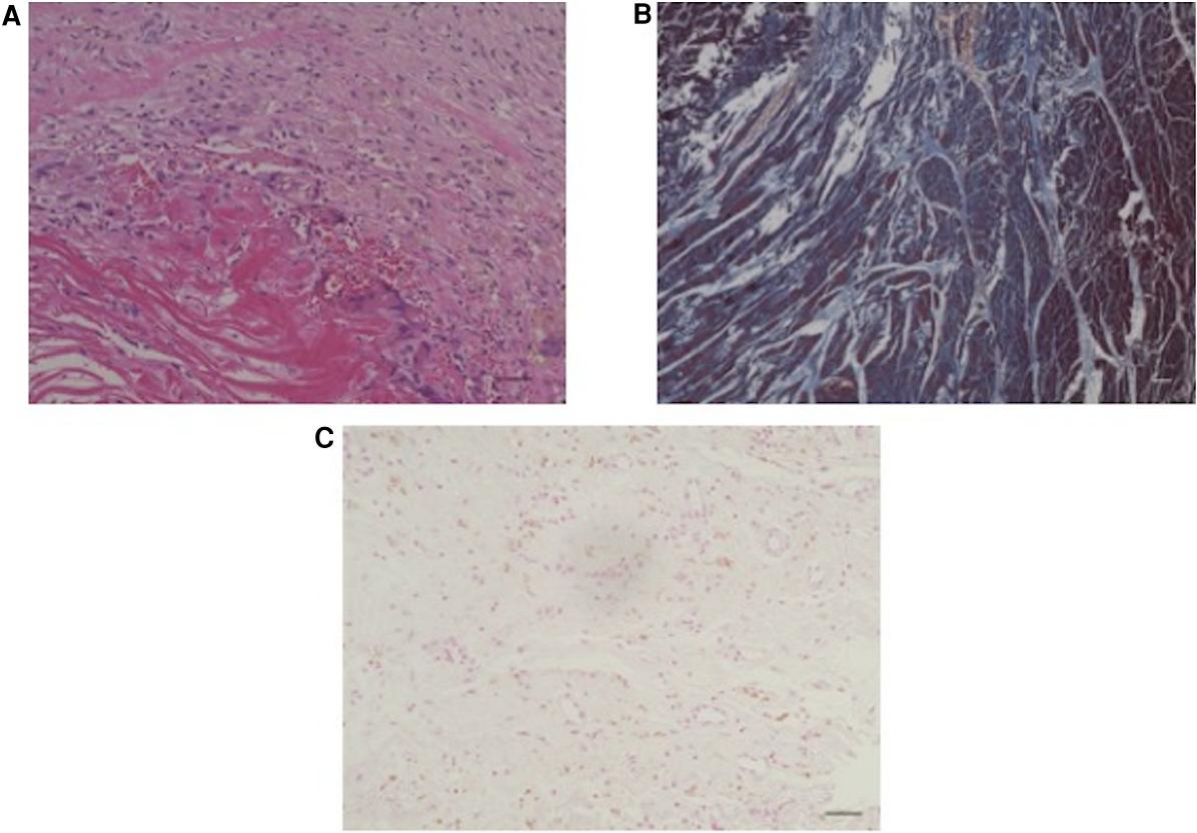
Case 1. A photomicrograph of the myocardium. (*A*) A high-power image of the myocardium demonstrates a significant infiltration of inflammatory cells (hematoxylin–eosin stain: magnification, ×200). (*B*) Interstitial fibrosis in the myocardium (Masson trichrome stain, ×100). (*C*) The special stain for calcium does not show obvious calcium deposition (von Kossa stain, ×200). Scale bars: 50 µm.

A 15-year-old boy with no significant medical history developed sudden cardiac arrest during long-distance running. He was transported to our hospital with refractory ventricular fibrillation. In our emergency unit, peripheral ECMO was commenced through the femoral vessels. The following day, the ECMO was converted to paracorporeal biventricular assist device (BiVAD) support using centrifugal pumps. After the ECMO decannulation, compartment syndrome developed in the lower extremities and fasciotomy was performed. The patient required RRT for 2 months for the treatment of acute kidney injury. One month after admission, his cardiac function improved with a left ventricular (LV) ejection fraction of 45%, and the BiVAD was successfully removed once; however, a repeat left ventricular assist device (LVAD) implantation was required because of the recurrence of ventricular arrhythmia. The patient was listed as a candidate for heart transplantation, and an implantable continuous-flow LVAD (Jarvik 2000; Jarvik Heart, Inc., New York, NY, USA) was placed 3 months after admission as a bridge-to-transplant. Before the Jarvik 2000 implantation, CT revealed myocardial calcification in both ventricles, which had not been detected upon admission (*Figure 1A*). He was discharged 8 months after admission. During hospitalization, his serum calcium level was within normal limits (6.4–8.9 mg/dL), and his serum phosphate level was slightly high (4.9–5.1 mg/dL). CT was repeated at 1 month (*Figure 1B*), 4 months (*Figure 1C*), and 11 months (*Figure 1D*) after the first CT, showing a gradual regression of the myocardial hyperdensities. The patient successfully underwent heart transplantation after a 3-year waiting period. A histological evaluation of the LV myocardium was carried out using a heart specimen obtained at the time of Jarvik 2000 implantation (*Figure 2A–C*). This evaluation revealed significant inflammatory cell infiltration and massive myocardial fibrosis, whereas calcium deposition was not obvious (*Figure 2A–C*).

### Case 2

**Figure 3:**
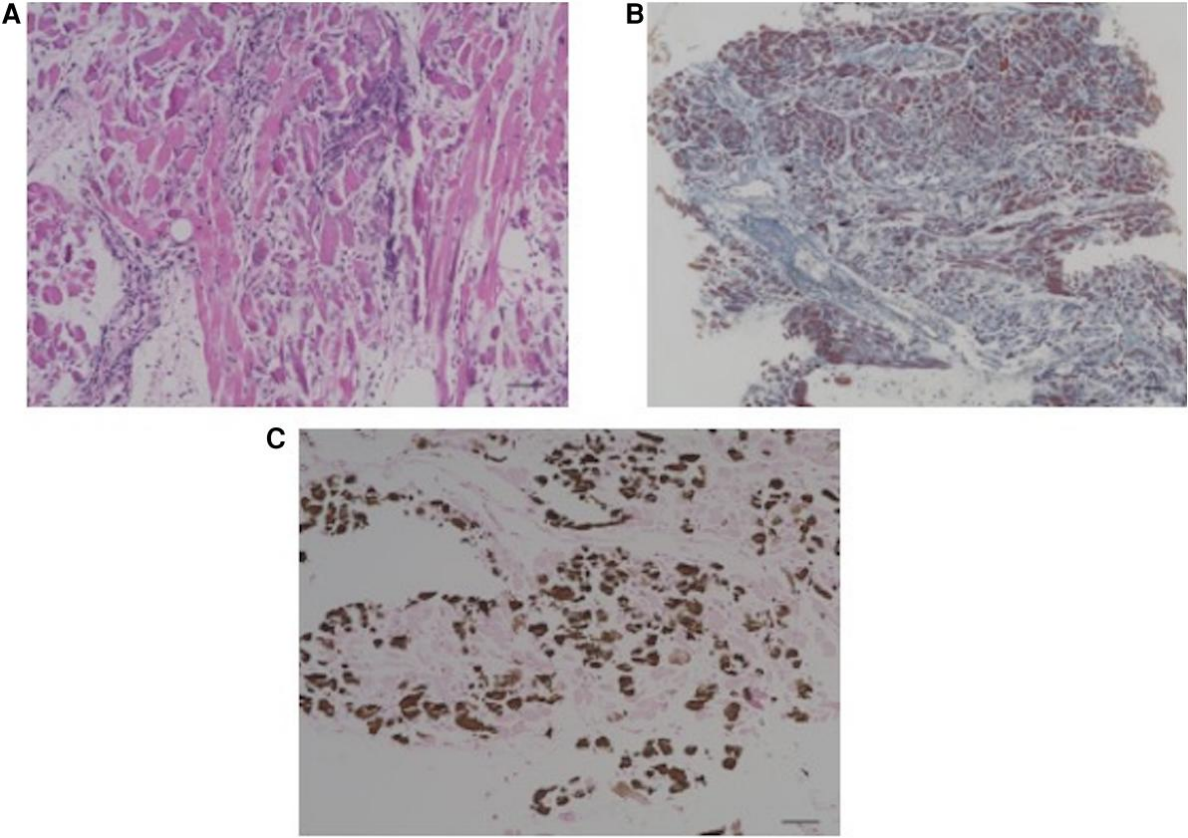
Case 2. A time course of myocardial calcification on CT (*A*) at admission and at (*B*) 2 months and (*C*) 5 months after admission. Biventricular myocardial calcification was detected at admission and became obvious during follow-up. Scale bars: 50 µm.

**Figure 4:**
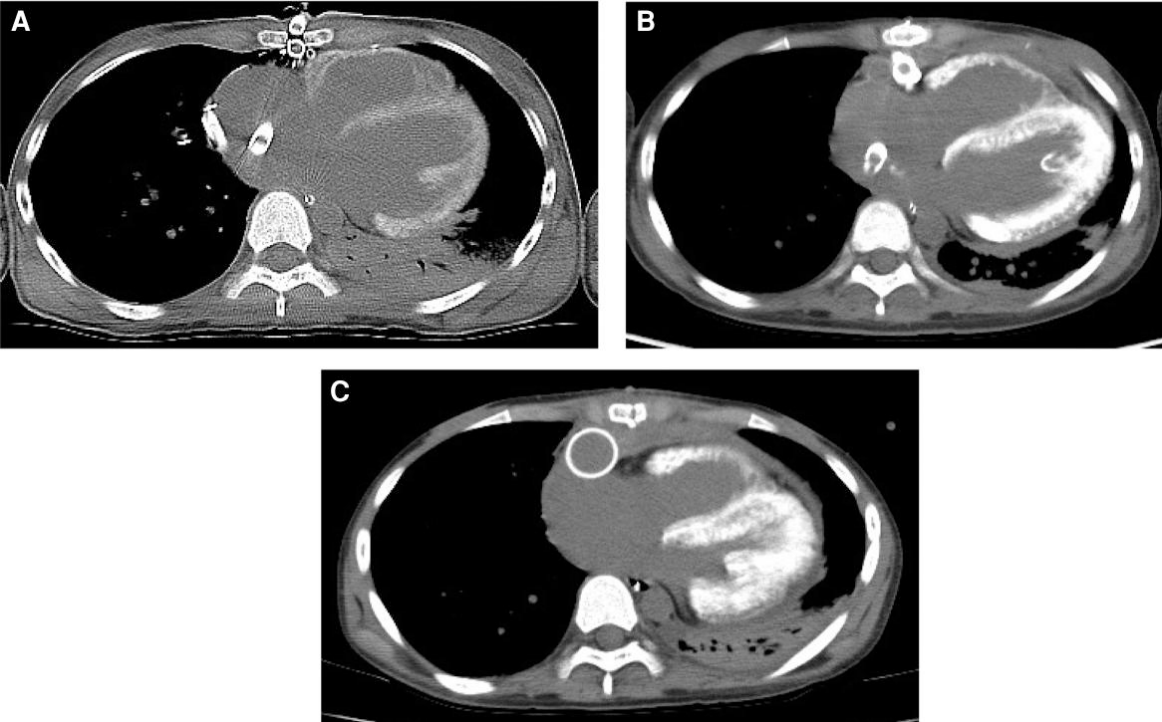
Case 2. A photomicrograph of the myocardium. (*A*) Ahigh-power image of the myocardium demonstrates a significant infiltration of inflammatory cells (hematoxylin–eosin stain: magnification, ×200). (*B*) Interstitial fibrosis in the myocardium (Masson trichrome stain, ×100). (*C*) Extensive calcium deposition in the myocardium (von Kossa stain, ×200). Scale bars: 50 µm.

**Figure 5:**
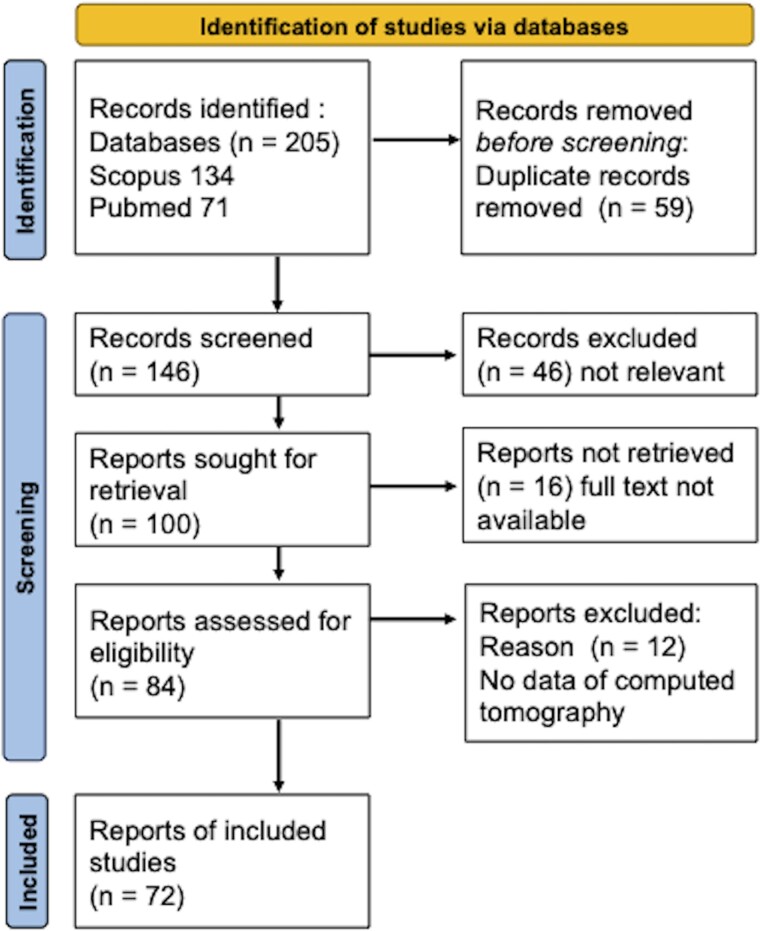
A flow diagram of search results and study selection.

A 13-year-old girl was admitted to an outside hospital with fever and abdominal pain. She was diagnosed with myocarditis, and emergent venoarterial ECMO was commenced through the neck vessels. The ECMO was converted to a central ECMO with right and left atrial drainage and ascending aortic cannulation. The patient was transferred to our hospital and she underwent BiVAD implantation with the use of a centrifugal pump. An LV specimen was obtained at the time of BiVAD implantation, and a histological evaluation showed a significant filtration of inflammatory cells, myocardial fibrosis, and significant calcium deposition in the myocardium (*Figure 3A–C*). CT showed myocardial calcification in both ventricles on admission (*Figure 4A*). Her serum calcium level remained normal (7.7–9.8 mL/dL), and her serum phosphate level was slightly elevated (6.1 mg/dL). She required 2 months of RRT for the treatment of acute kidney injury. Her cardiac function did not improve, and her LV ejection fraction was 30%. She was listed as a candidate for heart transplantation, and an implantable continuous-flow LVAD (Jarvik 2000) was placed 3 months after admission. The right ventricular assist device was successfully removed 1 month after the Jarvik 2000 implantation. CT was repeated at 2 months (*Figure 4B*) and 5 months (*Figure 4C*) after the first detection of myocardial calcification, and the calcification did not regress during follow-up. Although the patient was discharged 8 months after admission, she was readmitted to the hospital after 2 months because of malignant headache. A head CT revealed massive cerebral haemorrhage, and the patient died 2 weeks after the readmission.

## Systematic review

In total, 205 case reports were identified and reviewed. Seventy-two reports involving 75 patients met the inclusion criteria. *Figure 5* depicts a flowchart of the study selection process. All reported patients were categorized according to their associated clinical features: sepsis, myocarditis, and other pathologies, such as ischaemic heart disease and post-heart transplantation. Sepsis was present in 24 (32%) patients, myocarditis in 14 (18%), and other pathologies in 37 (50%). The serum calcium level was described in 21 reports, with only one reporting hypercalcaemia. *Table 1* contains a complete list of articles included in the review and provides a description of the patients according to their associated clinical features. *Table 2* details the patients’ characteristics, treatments, and outcomes by their associated clinical features.

**Table 1:** A breakdown of all cases

Author, year	Age, gender	Medical history	Clinical presentation	Calcification detection (days after admission)	ECMO	RRT	Outcome
**Sepsis-associated myocardial calcification**							
Lim A, 2021^4^	51, male	Urosepsis 9 years prior	Dyspnoea	12	No	No	Alive
Ahmed T, 2019^5^	21, female	Vaginal delivery 5 days prior	Abdominal pain, fever	19	Yes	Yes	Alive
Santos ACEZ, 2019^6^	15, male	SLE arthritis, fever	Myalgia	Postmortem	No	Yes	Death
Hibi A, 2018^7^	46, male	N.S.	Fatigue, fever	48	No	Yes	Death
Glick Y, 2020^8^	45, male	HIV positivity, hepatocellular carcinoma	Dyspnoea, fever	14	No	No	Death
Yokoi M, 2021^9^	54, male	Iliac bone graft surgery	General fatigue 10 days prior	10	No	No	Alive
Canu M, 2021^10^	52, male	Peritonitis following prostate surgery	Septic shock	10	No	No	Alive
Nijjar PS, 2019^11^	41, female	AML	Septic shock	N/A	No	No	Death
Chen YS, 2022^12^	26, female	Type 2 DM hypertension	Right frank pain, dysuria	7	No	Yes	Alive
Hu JY, 2020^13^	36, female	Systemic amyloidosis CRF on HD	Septic shock	1	No	No	Death
Kang MH, 2022^14^	51, male	Liver cirrhosis	General weakness	6	No	No	Alive
Li J, 2021^15^	34, female	After laparotomy CRF on HD	Septic shock	3	No	Yes	Death
Li J, 2021^15^	30, male	CRF on HD lower extremity	Open wounds in	1	No	Yes	Alive
Yoshihara S, 2021^16^	67, male	Liver cirrhosis, hepatocellular carcinoma	Septic shock	16	No	Yes	Alive
Monnier-Cholley L, 2018^17^	76, female	CLL	Septic shock	90	No	No	Alive
Siani A, 2022^18^	38, female	Wolfram syndrome	Pyelonephritis ARDS	1	No	No	Alive
Richard M, 2023^19^	51, female	Biliary cholangitis	Abdominal pain, fever	6	No	No	Alive
Chan WCS, 2016^20^	56, male	Post-abdominal surgery	Peritonitis	42	No	Yes	Death
Kapandji N, 2018^21^	66, male	N.S.	Bacteraemic pneumonia	32	Yes	Yes	Alive
Kapandji N, 2018^21^	26, female	Drug abuse	Septic shock	20	Yes	Yes	Death
Garcia-Cardenas M, 2023^22^	63, male	N.S.	Septic shock	N/A	No	No	Alive
Sedlock C, 2019^23^	56, female	AML	Vomiting	N/A	No	Yes	N/A
Torfs M, 2016^24^	56, female	N.S.	Respiratory failure	13	No	No	N/A
Ng R, 2018^25^	15, male	Kleinfelter	Septic shock	N/A	No	No	N/A
**Myocarditis-associated myocardial calcification**							
Washino M, 2020^26^	43, female	N.S.	Leg oedema, nephrotic syndrome	5	No	Yes	Alive
Sui ML, 2022^27^	17, male	N.S. chest tightness	Fever	10	Yes	Yes	Alive
Zeng C, 2023^28^	16, male	N.S. shock	Cardiogenic	10	Yes	Yes	Alive
Kimura Y, 2019^29^	15, male	N.S.	Flu-like symptom	30	Yes	No	Alive
Yang Z, 2022^30^	42, male	Myocarditis 1 month prior	Heart failure symptom	30	No	No	Alive
You B, 2023^31^	16, male	N.S.	Fever, cough	13	Yes	Yes	Alive
Koo CW, 2023^32^	61, male	N.S.	Cardiogenic shock	1	No	No	Death
Salaun E, 2023^33^	38, male	N.S. deterioration	Rapid clinical	14	No	Yes	Alive
Shoya K, 2023^34^	0, female	N.S.	Vomiting	32	Yes	No	Death
Díez-Delhoyo F, 2015^35^	32, male	HIV positive, myocarditis 2 months prior	Acute heart failure	60	No	No	Alive
Wittekind SG, 2017^36^	0, female	N.S.	Maternal HCV positivity	18	No	No	Alive
Tu X, 2020^37^	9, female	N.S.	Vomiting	14	No	Yes	Alive
Deng Y, 2023^38^	56, male	Myocarditis 10 years prior	Palpitation, shortness of breath	1	No	No	Alive
Pejic M, 2019^39^	78, female	Leukaemia	Progressive fatigue	125	No	No	N/A
**Myocardial calcification associated with other pathologic conditions**							
Chen W, 2022^40^	47, male	N.S. oliguria	Chest tightness,	60	No	Yes	Alive
Austin CO, 2013^41^	44, male	Liver and renal failure	Cardiogenic shock	2	No	No	Death
Deguchi K, 2016^42^	20, male	Biliary atresia, post-liver transplantation	Graft failure	N/A	No	No	Alive
Na JY, 2018^43^	53, male	Ischaemic heart disease, CRF on HD	Collapse in walkway	Postmortem	No	Yes	Death
Kim DY, 2022^44^	91, female	Old myocardial infarction	Dyspnoea	1	No	No	Alive
Minomo S, 2021^45^	24, male	N.S.	Fever, epigastric pain	47	No	Yes	Alive
Chango Azanza DX, 2021^46^	16, male	HIV positivity, dilated cardiomyopathy	Chest pain	N/A	No	No	Alive
Duarte SBCP, 2020^47^	33, female	Post-heart transplantation	Acute graft failure	N/A	No	Yes	Death
Dederer J, 2018^48^	62, female	Rib fracture, Pneumothorax	Acute heart failure	60	No	Yes	Alive
Mansour L, 2023^49^	74, male	N.S.	Transient ischaemic attack	1	No	No	Alive
Tominaga O, 2020^50^	71, female	Hypertension, dyslipidaemia	Palpitation	1	No	No	Alive
Tung RT, 2021^51^	70, male	Atrial fibrillation	Dyspnoea	N/A	No	No	Alive
Vural KM, 2021^52^	20, female	Rickets	Mitral stenosis	N/A	No	No	Alive
Ito S, 2015^53^	88, male	Hypertrophic cardiomyopathy	No symptom	N/A	No	No	Alive
Carande EJ, 2022^54^	51, female	Pseudoxanthoma elasticum	Ascites	18 months previously	No	No	Alive
Li J, 2021^15^	20, male	Trisomy 21, atrioventricular septal defect	COVID-19 pneumonia	40	Yes	Yes	Alive
Lippolis A, 2020^55^	74, female	Diabetes mellitus, atrial fibrillation	Dyspnoea	N/A	No	No	Alive
Seo KW, 2018^56^	54, female	Hyperparathyroidism, CRF on HD	Dyspnoea	N/A	No	No	Alive
Rodorigues RP, 2022^57^	35, male	ANK2 mutation	Chest discomfort	N/A	No	No	Alive
Takahashi H, 2013^58^	47, female	Hyperparathyroidism	Dyspnoea	17	No	No	Alive
Lin A, 2014^59^	23, male	Dilated cardiomyopathy, post-heart transplantation	Acute graft failure	22	No	Yes	Alive
Rosenthal JL, 2014^60^	64, male	Post-coronary artery bypass graft surgery	Acute vision loss	N/A	No	No	Alive
Marciniak A, 2015^61^	70, female	CLL	No symptom	N/A	No	No	Alive
Renilla A, 2015^62^	40, male	Post-pace maker implantation	Heart failure symptom	N/A	No	No	Alive
Buchner S, 2015^63^	65, male	Weber–Christian disease	Chest pain	N/A	No	No	Alive
Ananthakrishna R, 2016^64^	60, male	Myocardial infarction	Dyspnoea	N/A	No	No	Alive
Maemura S, 2016^65^	37, male	N.S.	Dyspnoea	N/A	No	No	Alive
Nikolaidou C, 2020^66^	69, female	Type 2 DM	Dyspnoea	N/A	No	No	Alive
Abraham-Foscolo MM, 2022^67^	72, male	Hypertension, diabetes mellitus	Fever,	N/A	No	No	Alive
Shako D, 2022^68^	72, male	Hypertension, diabetes mellitus	No symptom	N/A	No	No	Alive
Wert L, 2023^86^	76, male	Atrial fibrillation, hypertension	Dyspnoea	N/A	No	No	Alive
Sozzi FB, 2022^69^	81, female	Rheumatic fever	Acute chest pain	N/A	No	No	N/A
Hoang K, 2020^70^	67, female	Hypertension	Abdominal pain	N/A	No	No	N/A
Alani A, 2015^71^	94, male	Ischaemic cardiomyopathy	Shortness of breath	N/A	No	No	N/A
Mitsui K, 2021^72^	78, female	N.S.	Acute heart failure	N/A	No	No	Death
Yang CC, 2022^73^	78, female	CRF on HD diabetes mellitus	Shortness of breath	N/A	No	No	N/A
Tinoco M, 2021^74^	71, female	Hypertension	Syncope	N/A	No	No	N/A

**Table 2:** Patients’ characteristics, treatments, and outcomes

		Sepsis-related (*n* = 24)	Myocarditis-related (*n* = 14)	Other pathologies (*n* = 37)
Male	13 (54%)	9 (64%)	22 (59%)	
Age	49 (33–56)	24 (15–42)	65 (44–72)	
Left ventricular calcification	21 (87%)	9 (64%)	32 (86%)	
Right ventricular calcification	0 (0%)	0 (0%)	1 (3%)	
Biventricular calcification	3 (13%)	5 (36%)	4 (11%)	
Comorbidity				
Ischaemic heart disease	0 (0%)		0 (0%)	5 (13%)
End-stage renal disease	3 (12%)		0 (0%)	2 (6%)
Liver failure	2 (8%)		0 (0%)	2 (6%)
Leukaemia	3 (12%)		1 (7%)	0 (0%)
Treatment				
Intensive care unit	20 (83%)	11 (78%)	15 (40%)	
ECMO	3 (12%)	5 (35%)	1 (3%)	
Renal replacement therapy	11 (46%)	6 (43%)	7 (19%)	
Outcome				
Death	8 (33%)	2 (14%)	4 (11%)	

ECMO, extracorporeal membrane oxygenation.

## Sepsis-related myocardial calcification

The median age at presentation was 49 [inter-quartile (IQR), 33–56] years. Myocardial calcification was detected on CT at a median of 12 (IQR, 6–20) days after admission, whereas it was not detected on CT on admission in 12 patients. It was found only in the LV in 21 patients and in both the right ventricle and the LV in three. Pathological conditions potentially related to sepsis were end-stage renal disease in four patients, post-surgical condition in five, liver cirrhosis in two, and not specified in six. Eight patients were immunocompromised (human immunodeficiency virus positivity, systemic lupus erythematosus, diabetes mellitus, Wolfram syndrome, and drug abuse in one patient each and leukaemia in three patients). Eighteen patients developed shock due to sepsis and required inotropic support. RRT was required in 10 patients. Three patients were supported by ECMO for 6, 24, and 94 days, respectively. The following bacteria were identified from the blood culture of 12 patients: *Escherichia coli* in six, *Staphylococcus aureus* in three, *Neisseria meningitidis* in one, *Klebsiella pneumoniae* in one, and *Streptococcus pyogenes* in one. Eight patients died at a median 49 (IQR, 18–73) days, whereas 13 patients recovered and were discharged at a median of 83 (IQR 61–92) days after admission. Outcomes were not specified in three patients. Echocardiography data were described in 19 patients. Twelve patients showed reduced LV systolic function with an ejection fraction of <40% at a median of 3 (IQR, 1–6) days after admission. Recovery of cardiac function was described in three patients during the follow-up period, with normal LV systolic function in the three patients and mildly reduced function in one.

## Myocarditis-related myocardial calcification

The median age at presentation was 24 (IQR, 15–42) years. Ten patients were previously healthy with no history of heart disease. These patients developed acute circulatory failure within 3 days of admission and required intensive care treatment. ECMO support was required in five patients, and RRT was performed in six patients. One patient with acute myelogenous leukaemia developed palpitation and shortness of breath after the 3 weeks of anthracycline therapy. Although this patient did not experience circulatory failure, cardiovascular magnetic resonance showed irregular subepicardial enhancement, suggestive of myocardial inflammation. Myocardial calcification was found in the LV only in nine patients and in both the right ventricle and the LV in five, and it was detected on CT at a median of 14 (IQR, 10–30) days after admission. Two patients died at 83 and 39 days after admission, respectively. One patient underwent LVAD implantation followed by a heart transplantation 3 years after admission. Seven patients recovered and were discharged at a median of 38 (IQR, 30–60) days after admission. Outcomes were not described in one patient. Three patients had myocarditis 10 years, 2 months, and 1 month before admission, respectively. These patients presented with heart failure symptoms, and myocardial calcification was detected on admission upon CT. In contrast to the 10 patients described above, these three patients did not require intensive care treatment and were discharged with oral medication. The LV systolic function was significantly reduced in all patients on admission. A complete recovery of LV systolic function was reported in four patients at 2–18 months after discharge.

## Myocardial calcification associated with other pathological conditions

In total, 37 patients were reported to have myocardial calcification without sepsis or myocarditis. The myocardial calcification was found in the LV only in 32 patients, in both the right ventricle and the LV in 3, in the interventricular septum in 1, and in the right ventricular apex in 1. Fifteen patients had a severe clinical course requiring intensive care. Relevant medical history included ischaemic heart disease in two patients, heart transplantation in two, liver failure in two, valvular calcification in two, pulmonary embolism in one, COVID-19 pneumonia in one, Valsalva sinus rupture in one, and TAFRO syndrome in one. One patient with heart transplantation, two with liver failure, and one with acute heart failure without relevant medical history died despite intensive care treatment, whereas 10 patients recovered and were discharged. Outcomes were not specified in one patient. Twenty-two patients received conservative heart failure therapy. Twenty patients recovered without intensive care treatment, while outcomes were not specified in two patients. Relevant medical history included ischaemic heart disease in four patients, hyperparathyroidism in three, diabetes mellitus in three, endomyocardial fibrosis in two, valvular calcification in one, dilated cardiomyopathy in one, hypertrophic cardiomyopathy in one, leukaemia in one, pseudoxanthoma in one, ANK2 mutation in one, and no specific medical history in four.

## Time course of myocardial calcification

Time-course changes in the CT findings of myocardial calcification were reported in nine patients: six patients with myocarditis,^26–31^ two with sepsis,^4,5^ and one with Valsalva sinus rupture.^40^ More than 2 years of follow-up was reported in six patients, showing that the CT findings of myocardial calcification persisted but subsided over time. One patient underwent time-course cardiac magnetic resonance imaging which showed improved T1 mapping, extracellular fraction, and systolic function over time as well as the improvement in the calcification on CT.

## Autopsy findings of myocardial calcification

Autopsy was performed in 10 patients: four patients with sepsis,^6,7,13,21^ two with myocarditis,^29,32^ two with liver failure,^41,42^ one with ischaemic heart disease,^43^ and one with no relevant aetiology.^72^ Gross pathological examination showed myocardial discolouration in seven patients. Microscopic examination showed calcium deposition in both the cytoplasm and interstitium. Extensive interstitial fibrosis and collection of inflammatory cells were observed in patients with myocarditis, sepsis, and ischaemic heart disease, whereas no significant myocardial inflammation was found in patients with liver failure. Electron microscopy analysis in a patient with sepsis showed extensive myocytolysis and mitochondria crystolysis as well as calcification of the entire mitochondrial matrix.

## Discussion

The four main findings of this systematic review were as follows. First, although various medical conditions can cause myocardial calcification, accompanying conditions commonly reported with myocardial calcification were sepsis and myocarditis. Secondly, in our systematic review, 13 (54%) patients with sepsis and 11 (78%) in myocarditis survived and were discharged. Thirdly, the CT findings suggestive of myocardial calcification tend to regress over time, if the underlying disease can be treated. Fourthly, calcification deposition occurs in both the cytoplasm and interstitium. Calcification of the mitochondrial matrix has also been reported.

Myocardial calcification has been extensively documented and is classified into two types: dystrophic (calcium is deposited in areas of necrosis) and metastatic (associated with hypercalcaemic conditions such as renal disease and hyperparathyroidism).^75^ Cardiac dysfunction caused by sepsis, termed sepsis-induced cardiomyopathy, is thought to result from myocardial calcium dysregulation.^76^ Sepsis-induced alterations in calcium handling within cardiomyocytes have been extensively studied using animal models and include inhibition of L-type Ca^2+^ channels, inhibition of sarcoplasmic reticulum Ca^2+^ ATPase, increased ryanodine receptor Ca^2+^ leakage, and decreased myofilament Ca^2+^ sensitivity.^77–81^ Such dysregulation of Ca^2+^ homeostasis causes decreased uptake of Ca^2+^ and increased Ca^2+^ leakage from the sarcoplasmic reticulum, resulting in mitochondrial Ca^2+^ overload and leading to mitochondrial dysfunction. Mitochondrial dysfunction causes an increase in reactive oxygen species and accelerates Ca^2+^ overload in cardiac cells.^82,83^ Given that mitochondrial dysfunction and increased oxidative stress are associated with various types of pathological conditions, such as myocarditis and ischaemic heart disease, these factors may play a crucial role in the progression of dystrophic myocardial calcification.

Metastatic tissue calcification is defined as calcium deposition in formally healthy tissues secondary to high serum levels of calcium and phosphate. In 1967, Berlyne and Shaw^84^ described 15 patients with severe renal failure and metastatic conjunctival calcification. They suggested that serum Ca × P product of >70 mg^2^/dL^2^ was responsible for passive precipitation in metastatic calcification. End-stage renal disease is the most commonly recognized cause of metastatic myocardial calcification, leading to hyperphosphataemia, vitamin D deficiency, secondary hyperparathyroidism, and hypercalcaemia.^85^ In this systematic review, 24 (32%) patients required RRT (5 patients with end-stage renal failure and 19 patients with acute renal failure). Renal failure appears to have a major influence on myocardial calcification.

Although this systematic review was unable to determine effective treatments for myocardial calcification, a successful management of associated pathologies can result in the recovery of myocardial function and regression of myocardial calcification. The mortality rate of myocardial calcification associated with sepsis and myocarditis is high, which may be due to the severity of the patients’ general condition. The CT findings of myocardial calcification can regress over time and is not necessarily a fatal condition. Mitochondrial dysfunction and increased oxidative stress might be therapeutic targets, and strict controls of the serum calcium and phosphonate levels are needed. In the setting of severe circulatory failure, prompt initiation of VAD support can bridge the patients to heart transplant candidacy, as shown in our two patient cases. Myocardial calcification is thought to be a serious end stage of various pathologies and a sign of poor prognosis. However, the development of intensive care management and a greater understanding of cardiac calcium metabolism can improve the outcome of patients with myocardial calcification. We believe that this systematic review will provide an opportunity to recognize the pathological condition underlying myocardial calcification and will lead to further research that will improve treatment outcomes.

## Supplementary data

Supplementary data are available at *European Heart Journal – Imaging Methods and Practice* online.

## Supplementary Material

qyae079_Supplementary_Data

## Data Availability

The data sets used and/or analysed during the current study are available from the corresponding author on reasonable request.

## Lead author biography

Dr Takashi Kido received his MD degree from the Osaka University in 2007. He completed his training at Osaka Women's and Children's Hospital, Osaka City General Hospital, and the National Cerebral and Cardiovascular Center. After completing his PhD at Osaka University, he studied at Boston Children's Hospital and German Heart Center Munich as a postdoctoral research fellow and visiting physician. He is currently working as an assistant professor at the Department of Cardiovascular Surgery at Osaka University. His clinical research area includes congenital heart surgery and heart transplantation. His basic research aims to develop a new treatment approach based on metabolic and structural changes that causes heart failure.
