# Supplementary material for: Myocardial calcification: case reports and a systematic review
Source: Eur Heart J Imaging Methods Pract. 2024 Jul 30;2(3):qyae079. doi: 10.1093/ehjimp/qyae079 (PMC11367960; doi:10.1093/ehjimp/qyae079)
Supplement: qyae079_Supplementary_Data [file qyae079_supplementary_data.docx]

Data S1.

Publication date:

2013/1/1 ~ 2023/10/18

**Pubmed**

Case reports

(((“myocardial calcification"[Title/Abstract]) OR ("ventricular calcification"[Title/Abstract]))

OR ((myocardium[Title/Abstract]) AND (calcification[Title/Abstract]))

**Scopus**

Case reports

(Title-ABS-KEY ("myocardial calcification" OR "ventricular calcification"))

OR ((Title-ABS-KEY (myocardium) AND (Title-ABS-KEY (calcification))
